# Quantum Computation under Micromotion in a Planar Ion Crystal

**DOI:** 10.1038/srep08555

**Published:** 2015-02-25

**Authors:** S.-T. Wang, C. Shen, L.-M. Duan

**Affiliations:** 1 Department of Physics, University of Michigan, Ann Arbor, Michigan 48109, USA; 2 Center for Quantum Information, IIIS, Tsinghua University, Beijing 100084, PR China; 3 Department of Applied Physics, Yale University, New Haven, Connecticut 06511, USA

## Abstract

We propose a scheme to realize scalable quantum computation in a planar ion crystal confined by a Paul trap. We show that the inevitable in-plane micromotion affects the gate design via three separate effects: renormalization of the equilibrium positions, coupling to the transverse motional modes, and amplitude modulation in the addressing beam. We demonstrate that all of these effects can be taken into account and high-fidelity gates are possible in the presence of micromotion. This proposal opens the prospect to realize large-scale fault-tolerant quantum computation within a single Paul trap.

Scalable quantum computation constitutes one of the ultimate goals in modern physics^1^,^2^. Towards that goal, trapped atomic ions are hailed as one of the most promising platforms for the eventual realization^3^,^4^. The linear Paul trap with an one-dimensional (1D) ion crystal was among the first to perform quantum logic gates^5^,^6^,^7^ and to generate entangled states^8^,^9^,^10^, but in terms of scalability, the 1D geometry limits the number of ions that can be successfully trapped^11^,^12^. Another shortcoming of the 1D architecture is that the error threshold for fault-tolerant quantum computation with short-range gates is exceptionally low and very hard to be met experimentally^13^,^14^,^15^.

Generic ion traps, on the other hand, could confine up to millions of ions with a 2D or 3D structure^16^,^17^,^18^. More crucially, large scale fault-tolerant quantum computation can be performed with a high error threshold, in the order of a percent level, with just nearest neighbor (NN) quantum gates^19^,^20^,^21^,^22^. This makes 2D or 3D ion crystals especially desirable for scalable quantum computation. Various 2D architectures have been proposed, including microtrap arrays^23^, Penning traps^16^,^24^,^25^,^26^, and multizone trap arrays^27^,^28^. However, the ion separation distance in microtraps and penning traps is typically too large for fast quantum gates since the effective ion-qubit interaction scales down rapidly with the distance. In addition, fast rotation of the ion crystal in the Penning trap makes the individual addressing of qubits very demanding. Distinct from these challenges, Paul traps provide strong confinement; however, they are hampered by the micromotion problem: fast micromotion caused by the driving radio-frequency (rf) field cannot be laser cooled. It may thus create motion of large amplitudes well beyond the Lamb-Dicke regime^29^,^30^, which becomes a serious impediment to high-fidelity quantum gates.

In this paper, we propose a scheme for scalable quantum computation with a 2D ion crystal in a quadrupole Paul trap. We have shown recently that micromotion may not be an obstacle for design of high-fidelity gates for the two-ion case^31^. Here, we extend this idea and show that micromotion can be explicitly taken into account in the design of quantum gates in a large ion crystal. This hence clears the critical hurdle and put Paul traps as a viable architecture to realize scalable quantum computation. In such a trap, DC and AC electrode voltages can be adjusted so that a planar ion crystal is formed with a strong trapping potential in the axial direction. In-plane micromotion is significant, but essentially no transverse micromotion is excited due to negligible displacement from the axial plane. We perform gates mediated by transverse motional modes and show that the in-plane micromotion influences the gate design through three separate ways: (1) It renormalizes the average positions of each ion compared to the static pseudopotential equilibrium positions. (2) It couples to and modifies the transverse motional modes. (3) It causes amplitude modulation in the addressing beam. In contrast to thermal motion, the fluctuation induced by micromotion is coherent and can be taken into account explicitly. Several other works also studied the effect of micromotion on equilibrium ion positions and motional modes^32^,^33^,^34^, or used transverse modes in an oblate Paul trap to minimize the micromotion effect^35^. Here, by using multiple-segment laser pulses^36^,^37^,^38^, we demonstrate that high-fidelity quantum gates can be achieved even in the presence of significant micromotion and even when many motional modes are excited. Our work therefore shows the feasibility of quadrupole Paul traps in performing large scale quantum computation, which may drive substantial experimental progress.

A generic quadrupole Paul trap can be formed by electrodes with a hyperbolic cross-section. The trap potential can be written as Φ(*x*, *y*, *z*) = Φ_DC_(*x*, *y*, *z*) + Φ_AC_(*x*, *y*, *z*), where 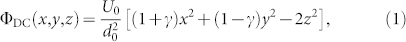

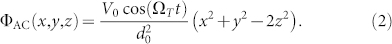


It contains both a DC and an AC part, with *U*_0_ being the DC voltage, and *V*_0_ being the AC voltage forming an electric field oscillating at the radiofrequency Ω*_T_*. The parameter *d*_0_ characterizes the size of the trap and *γ* controls the anisotropy of the potential in the *x*-*y* plane. We choose *γ* to deviate slightly from zero, so that the crystal cannot rotate freely in the plane, i.e. to remove the gapless rotational mode. The AC part, on the contrary, is chosen to be isotropic in the *x*-*y* plane. We let *U*_0_ < 0 such that the trapping is enhanced along the *z* direction in order to form a 2D crystal in the *x*-*y* plane. Disregarding the Coulomb potential first, the equations of motion of ions in such a trap can be written in the standard form of Mathieu equations along each direction: 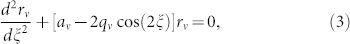
where *ν* ∈ {*x*, *y*, *z*}, and the dimensionless parameters are *ξ* = Ω*_T_t*/2, 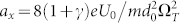, 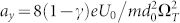, 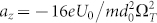, 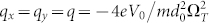, *q_z_* = −2*q*. Neglecting micromotion, one could approximate the potential as a time-independent harmonic pseudopotential with secular trapping frequencies *ω_ν_* = *β_ν_*Ω*_T_*/2, with 
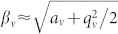
 being the characteristic exponents of the Mathieu equations^39^,^40^.

## Results

### Dynamic ion positions

Adding Coulomb interactions back, the static equilibrium positions can be found by minimizing the total pseudopotential^25^,^41^, or use molecular dynamics simulation with added dissipation, which imitates the cooling process in experiment^42^,^43^. In our numerical simulation, we start with *N* = 127 ions forming equilateral triangles in a 2D hexagonal structure. We then solve the equations of motion with a small frictional force to find the equilibrium positions 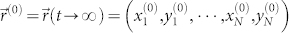, which is the starting point for the expansion of the Coulomb potential. Micromotion is subsequently incorporated by solving the decoupled driven Mathieu equations (see supplementary materials). The average ion positions 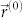 are found self-consistently, which differ slightly from the pseudopotential equilibrium positions (an average of 0.03 µm shift). Dynamic ion positions 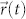 can be expanded successively as 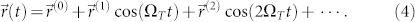
Numerically, we found that 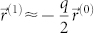 and 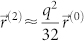, where the expression for 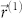 is consistent with previous results^31^,^32^,^42^. Micromotion thus only results in breathing oscillations about the average positions.

**Figure 1:**
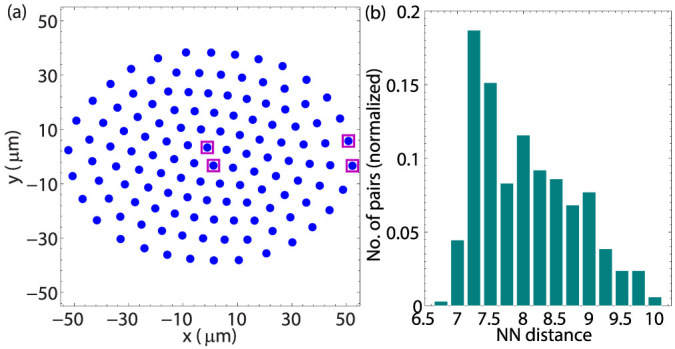
Crystal structure and distance distribution. (a) Average positions 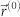 of 127 ions in a planar crystal. Breathing oscillations about these average positions occur due to micromotion. Two pairs of ions (enclosed in squares), one pair in the center and one near the edge, are used for the demonstration of a quantum gate later. (b) The distribution of nearest neighbor (NN) distance. The minimum, maximum, and average NN distances are 6.9 *µ*m, 10 *µ*m and 8.0 *µ*m respectively. Parameters used are: the number of ions *N* = 127; DC and AC potential *U*_0_ = −1.1 V, *V*_0_ = 90 V; AC rf frequency Ω*_T_*/2*π* = 50 MHz; the characteristic electrode size *d*_0_ = 200 *µ*m; ion mass *m* = 171*u* (*u* is the atomic mass unit) corresponds to ^171^Yb^+^ ion; the anisotropy parameter *γ* = 0.01; corresponding Mathieu parameters are *a_x_* ≈ −1.27 × 10^−3^, *a_y_* ≈ −1.25 × 10^−3^, *a_z_* ≈ 2.52 × 10^−3^, *q* ≈ −0.051, with respective secular trap frequencies *ω_x_*/2*π* ≈ 0.18 MHz, *ω_y_*/2*π* ≈ 0.22 MHz, *ω_z_*/2*π* ≈ 2.21 MHz; *ω_z_*/*ω_x,y_* > 10 ensures a planar crystal is formed.

Fig. 1(a) shows the average ion positions 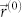 in the planar crystal. The distribution of NN distance is plotted in figure 1(b). We choose the voltages *U*_0_ and *V*_0_ such that the ion distance is kept between 6.5 *µ*m and 10 *µ*m. This ensures that crosstalk errors due to the Gaussian profile of the addressing beam are negligible, at the same time maintaining strong interaction between the ions. As micromotion yields breathing oscillations, the further away the ion is from the trap center, the larger the amplitude of micromotion becomes. With the furthest ion around 52 *µ*m from the trap center, the amplitude of micromotion is −*q*/2 × 52 ≈ 1.4 *µ*m, which is well below the separation distance between the ions but larger than the optical wavelength (see supplementary materials for the distribution of the amplitude of micromotion).

### Normal modes in the transverse direction

With the knowledge of ion motion in the *x*-*y* plane, we proceed to find the normal modes and quantize the motion along the transverse (*z*) direction. As ions are confined in the plane, micromotion along the transverse direction is negligible. The harmonic pseudopotential approximation is therefore legitimate. Expanding the Coulomb potential to second order, we have 
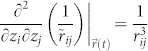
, where 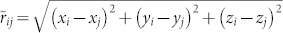 is the 3D distance and 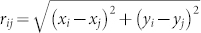 is the planar distance between ions *i* and *j*. To the second order, transverse and in-plane normal modes are decoupled. Note that coupling between the in-plane micromotion and the transverse normal modes has been taken into account in this expansion as the Coulomb potential is expanded around the dynamic ion positions 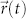. With significant in-plane micromotion, distances between ions are time-dependent, which in turn affects the transverse modes. We can expand the quadratic coefficients in series: 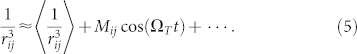
The time-averaged coefficients 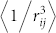 can be used to compute the transverse normal modes. The next order containing cos(Ω*_T_t*) terms can be considered as a time-dependent perturbation to the Hamiltonian. It contributes on the order of 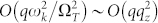 in the rotating wave approximation, where *ω_k_* is the transverse mode frequency. The term 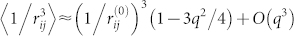, where 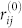 is the ion distance computed with 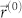 without considering micromotion (see supplementary materials). Here, the micromotion effect is an overall renormalization in the term 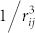, so it does not modify the normal mode structure. Instead, it slightly shifts down the transverse mode frequencies (in the order of *O*(*q*^2^)). Numerically, we found an average reduction of around 0.4 kHz in each transverse mode frequency with our chosen parameters. Although mode structure is not altered by this overall renormalization, the discrepancy in equilibrium positions compared to the pseudopotential approximation will modify both the normal mode structure and mode frequencies.

### High-fidelity quantum gates

**Figure 2:**
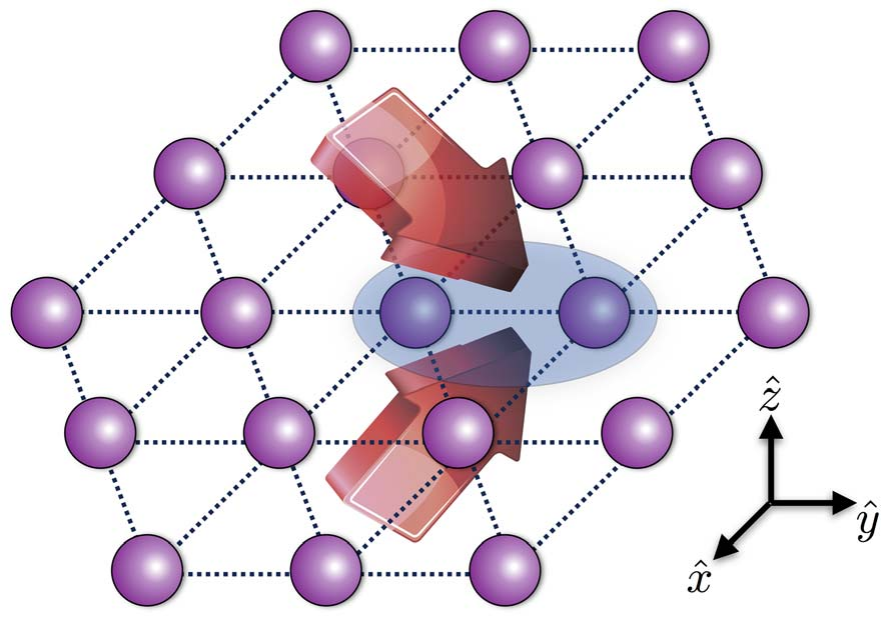
Nearest neighbor quantum gate in a 2D planar crystal. Two laser beams with a wave vector difference Δ*k* aligned in the *z* direction exert a spin-dependent force on the neighboring ions. Parameters used are: The wave vector difference of addressing beams Δ*k* = 8 *µ*m^−1^; Laser beams are assumed to take a Gaussian profile with a beam waist *w* = 3 *µ*m centered at the average positions of the respective ion; The Lamb-Dicke parameter 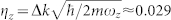. Other parameters are the same as in Fig. 1.

After obtaining the correct transverse normal modes, we now show how to design high-fidelity quantum gates with in-plane micromotion. Since NN gates are sufficient for fault-tolerant quantum computation in a planar crystal, we show as a demonstration that high-fidelity entangling gates can be achieved with a pair of NN ions in the trap center and near the trap edge. One may perform the gate along the transverse direction by shining two laser beams on the two NN ions with wave vector difference 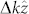 and frequency difference *µ* (see Fig. 2)^38^,^44^. The laser-ion interaction Hamiltonian is^37^ 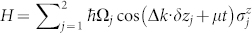, where Ω*_j_* is the (real) Raman Rabi frequency for the *j*th ion, 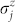 is the Pauli-*Z* matrix acting on the pseudospin space of internal atomic states of the ion *j*, and *δz_j_* is the ion displacement from the equilibrium position. Quantize the ion motion, 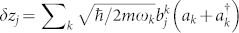, with 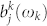 being the mode vector (frequency) for mode *k* and 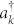 creates the *k*-th phonon mode. Expanding the cosine term and ignoring the single-bit operation, the Hamiltonian can be written in the interaction picture as 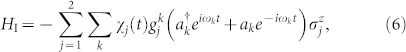
where 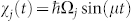, 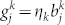 and the Lamb-Dicke parameter 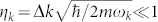. The evolution operator corresponding to the Hamiltonian *H*_I_ can be written as^37^,^38^,^45^

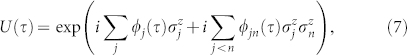
where the qubit-motion coupling term 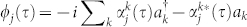 with 
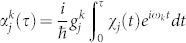
 and the two-qubit conditional phase 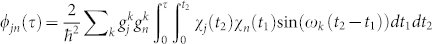. To realize a conditional phase flip (CPF) gate between ions *j* and *n*, we require 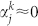 so that the spin and phonons are almost disentangled at the end of the gate, and also *ϕ_jn_*(*τ*) = *π*/4. It is worthwhile to note that in deriving Eq. (7), we dropped single-qubit operations as we are interested in the CPF gate. These fixed single-qubit operations can be explicitly compensated in experiment by subsequent rotations of single spins. (see supplementary materials for more detailed derivation and analysis).

As the number of ions increases, transverse phonon modes become very close to each other in frequencies. During typical gate time, many motional modes will be excited. We use multiple-segment pulses to achieve a high-fidelity gate^36^,^37^. The total gate time is divided into *m* equal-time segments, and the Rabi frequency takes the form 
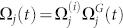
, with 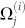 being the controllable and constant amplitude for the *i*th segment ((*i* − 1)*τ*/*m* ≤ *t* < *iτ*/*m*). Due to the in-plane micromotion, the laser profile 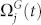 seen by the ion is time-dependent. In our calculation, we assume the Raman beam to take a Gaussian form, with 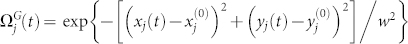, where *w* is the beam waist and 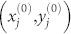 are the average positions for the *j*th ion. Any other beam profile can be similarly incorporated.

**Figure 3:**
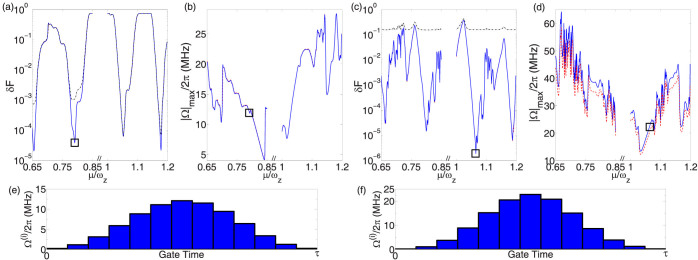
Gate infidelity and pulse shaping. (a), (b), and (e) are respectively the gate infidelity, the maximum Rabi frequency, and the thirteen-segment pulse pattern corresponding to the results marked by squares, for the center pair as labeled in Fig. 1. (c), (d), and (f) are the corresponding plots for the edge pair. The blue solid lines and the pulse sequences indicate the optimal results with micromotion considered. The red dashed lines are results for a genuine static harmonic trap without micromotion. Black dash-dot lines in (a) and (c) are obtained by applying the optimal solution for a static trap to the case with micromotion. All transverse modes are distributed between 0.85*ω_z_* and *ω_z_*. We optimize the gate near either end of the spectrum. The optimal results marked by the squares are *δF* = 4 × 10^−5^ and |Ω|_max_/2*π* = 12MHz (*δF* = 4 × 10^−6^ and |Ω|_max_/2*π* = 22 MHz) for the center (edge) pair. Parameters used are: total gate time *τ* = 50 × 2*π*/*ω_z_* ≈ 23 *µ*s; *m* = 13 segments are used; Doppler temperature 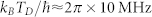 is assumed for all phonon modes. Other parameters are the same as in Fig. 1 and Fig. 2.

To gauge the quality of the gate, we use a typical initial state for the ion spin 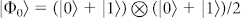 and the thermal state *ρ_m_* for the phonon modes at the Doppler temperature. The fidelity is defined as 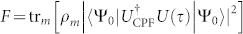 tracing over the phonon modes, with the evolution operator *U*(*τ*) and the perfect CPF gate 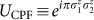. For simplicity, we take 
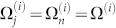
 for the ions *j* and *n*. For any given detuning *µ* and gate time *τ*, we optimize the control parameters Ω^(*i*)^ to get the maximum fidelity *F*. Fig. 3 shows the gate infidelity *δF* = 1 − *F* and the maximum Rabi frequency |Ω|_max_ = max*_i_* Ω^(*i*)^ for the center pair [(a) and (b)] and the edge pair [(c) and (d)] with 13 segments and a relatively fast gate *τ* ≈ 23 *µ*s. Detuning *µ* can be used as an adjusting parameter in experiment to find the optimal results. All transverse phonon modes are distributed between 0.85*ω_z_* and *ω_z_*. We optimize the gate near either end of the spectrum since optimal results typically occur there. Blue solid lines indicate the optimal results with micromotion and red dashed lines show the results for a genuine static harmonic trap, which are almost identical in (a), (b) and (c). It implies that micromotion can almost be completely compensated, but with a stronger laser power for the edge pair. If we apply the optimal result for the static trap to the realistic case with micromotion, the fidelity will be lower as indicated by the black dash-dot lines. This is especially so for the edge pair, where the fidelity is lower than 85% at any detuning. It is therefore critical to properly include the effect of micromotion. With corrected pulse sequences, a fidelity *F* > 99.99% can be attained with |Ω|_max_/2*π* ≈ 12 MHz (|Ω|_max_/2*π* ≈ 22 MHz) for the center (edge) ions. The Rabi frequencies can be further reduced by a slower gate and/or more pulse segments.

### Noise estimation

Micromotion of any amplitude does not induce errors to the gates as it has been completely compensated in our gate design. We now estimate various other sources of noise for gate implementation. In considering the effect of in-plane micromotion to the transverse modes, we are accurate to the order of *q*^2^, so an error of *q*^3^ ≈ 10^−4^ is incurred. The actual error is smaller since the Coulomb potential is an order of magnitude smaller than the trapping potential along the transverse direction. The cross-talk error probability due to beam spillover is 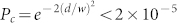, with the ion distance *d* ≳7 *µ*m and the beam waist *w* = 3 *µ*m. At the Doppler temperature 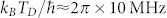, thermal spread in positions may degrade the gate fidelity. Similar to micromotion, thermal motion causes the effective Rabi frequency to fluctuate. With *ω_x,y_*/2*π* ≈ 0.2 MHz, there is a mean phonon number 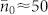 in the *x*-*y* plane. It gives rise to thermal motion with average fluctuation in positions, *δr* ≈ 0.23 *µ*m, which can be estimated as in Ref. 46. The resultant gate infidelity is *δF*_1_ ≈ (*π*^2^/4)(*δr*/*w*)^4^ ≈ 10^−4^. Lastly, we estimate the infidelity caused by higher-order expansion in the Lamb-Dicke parameter. The infidelity is 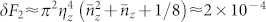, where 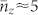 is the mean phonon number in the transverse direction^37^. Other than the effects considered above, micromotion may also lead to rf heating when it is coupled to thermal motion. However, simulation has shown that at low temperature *T* < 10mK and small *q* parameters, rf heating is negligible^42^,^47^. Heating effect due to rf phase shift and voltage fluctuation should also be negligible when they are well-controlled^42^.

## Discussion

It is worthwhile to point out that although we have demonstrated the feasibility of our gate design via a single case with *N* = 127 ions, the proposed scheme scales for larger crystals. The intuition is that through optimization of the segmented pulses, all phonon modes are nearly disentangled from the quantum qubits at the end of the gate. However, as the number of ions further increases, one would presumably need more and more precise control for all the experimental parameters (< 1% fluctuation in voltage for example). rf heating may also destabilize a much larger crystal^48^, and more careful studies are necessary for larger crystals.

One may also notice that in Ref. 31, we considered gates mediated by the longitudinal phonon modes, so the effect of micromotion is a phase modulation. Here, we utilize transverse modes so the amplitude of the laser beam is modulated. There are a few advantages in using the transverse modes: first, it is experimentally easier to access the transverse phonon modes in a planar ion crystal; second, in a planar crystal, the transverse direction is tightly trapped, so micromotion along that direction can be neglected; third, the transverse phonon modes do not couple to the in-plane modes and the in-plane micromotion affects the transverse modes via the time-dependence of the equilibrium positions, the effect of which is again suppressed due to tight trapping in the transverse direction.

In summary, we have demonstrated that a planar ion crystal in a quadrupole Paul trap is a promising platform to realize scalable quantum computation when micromotion is taken into account explicitly. We show that the in-plane micromotion comes into play through three separate effects, and each of them can be resolved. This paves a new pathway for large-scale trapped-ion quantum computation.

## Author Contributions

C.S. and L.-M.D. conceived the idea. S.-T.W. and C.S. carried out the calculations. S.-T.W. and L.-M.D. wrote the manuscript. All authors contributed to the discussion of the project and revision of the manuscript.

## Supplementary Material

Supplementary Information Supplementary Information: Quantum Computation under Micromotion in a Planar Ion Crystal
