# Supplementary material for: Quantum Computation under Micromotion in a Planar Ion Crystal
Source: Sci Rep. 2015 Feb 25;5:8555. doi: 10.1038/srep08555 (PMC4339801; doi:10.1038/srep08555)
Supplement: Supplementary Information — Quantum Computation under Micromotion in a Planar Ion Crystal [file srep08555-s1.pdf]

Supplementary Information: Quantum Computation under Micromotion in a Planar Ion Crystal

S.-T. Wang,[1,2] C. Shen,[1,3] and L.-M. Duan[1,2]

[1]*Department of Physics, University of Michigan, Ann Arbor, Michigan 48109, USA*
[2]*Center for Quantum Information, IIIS, Tsinghua University, Beijing 100084, PR China*
[3]*Department of Applied Physics, Yale University, New Haven, Connecticut 06511, USA*

In this supplementary information, we provide more details on the iterative method to find dynamic ion positions, and also consider the effect of in-plane micromotion to the transverse normal modes. We also include a more detailed derivation for the Hamiltonian and time-evolution operator for a two-ion entangling gate.

I. ITERATIVE METHOD TO FIND DYNAMIC ION POSITIONS

As discussed in the main text, the equations of motion in each direction can be written in the standard form of Mathieu equations (neglecting Coulomb potential):

$$\frac{d^2 r_\nu}{d\xi^2} + [a_\nu - 2q_\nu \cos(2\xi)]\, r_\nu = 0, \tag{1}$$

where $\nu \in \{x, y, z\}$, $\xi = \Omega_T t/2$, and dimensionless parameters a_ν and q_ν are defined in the main text. The characteristic exponents β_ν can be computed from a_ν and q_ν iteratively[1]. A pseudopotential can then be obtained with secular frequencies $\omega_\nu = \beta_\nu \Omega_T/2$ and

$$e\left(\Phi_{\rm DC} + \Phi_{\rm AC}\right) \approx \frac{1}{2}m\omega_x^2 x^2 + \frac{1}{2}m\omega_y^2 y^2 + \frac{1}{2}m\omega_z^2 z^2. \tag{2}$$

Assuming tight trapping along the z direction, i.e. $\omega_z/\omega_{x,y} > 10$, a planar crystal is formed in the x-y plane. Adding the Coulomb potential V_C, one acquires a time-independent potential in the plane:

$$V_{\rm pseudo}(x,\, y) = \sum_i \left(\frac{1}{2}m\omega_x^2 x_i^2 + \frac{1}{2}m\omega_y^2 y_i^2\right) + \sum_{i<j} \frac{e^2}{4\pi\epsilon_0\sqrt{(x_i - x_j)^2 + (y_i - y_j)^2}}. \tag{3}$$

$i = 1, 2, \cdots, N$, where N is the number of ions. Numerically, we start with $N = 127$ ions forming equilateral triangles in a 2D hexagonal structure [Fig. 1(a)], and find the static equilibrium positions $\vec{r}^{\,(0)} = (x_1^{(0)}, y_1^{(0)}, \cdots, x_N^{(0)}, y_N^{(0)})$ under this pseudopotential approximation by solving the classical equations of motion with a frictional force $(-\eta(\dot{x} + \dot{y}))$, simulating the cooling process in experiment. This set of static equilibrium positions [marked by squares in Fig. 1(b)] is the starting point to derive the oscillatory behavior of each ion under micromotion.

In a planar crystal, the ions oscillate slightly around their average positions, so it is appropriate to expand the Coulomb potential around the equilibrium positions $\vec{r}^{\,(0)}$. To the second order, the Coulomb potential can be written in a quadratic form:

$$V_C \approx \frac{1}{2}\vec{r}^{\,T} M_C \vec{r} + \vec{g}^{\,T}\vec{r} + \text{constant term}, \tag{4}$$

where $\vec{r} = (x_1, y_1, \cdots, x_N, y_N)$, M_C is a $2N \times 2N$ matrix, and $\vec{g}$ is a $2N$-vector. The trapping potential can also be written in this coordinate basis:

$$e\left(\Phi_{\rm DC} + \Phi_{\rm AC}\right) = \frac{1}{2}\vec{r}^{\,T} M_{DC}\vec{r} + \frac{V_0}{d_0^2}\cos(\Omega_T t)\vec{r}^{\,T} I_{2N}\,\vec{r}, \tag{5}$$

where I_{2N} is the $2N \times 2N$ identity matrix, and M_{DC} is a diagonal matrix with $2(1+\gamma)eU_0/d_0^2$ in the odd rows (x coordinates), and $2(1-\gamma)eU_0/d_0^2$ in the even rows (y coordinates). Therefore, the total potential energy is

$$V = \frac{1}{2}\vec{r}^{\,T}(M_{DC} + M_C)\vec{r} + \frac{V_0}{d_0^2}\cos(\Omega_T t)\vec{r}^{\,T} I_{2N}\,\vec{r} + \vec{g}^{\,T}\vec{r}. \tag{6}$$

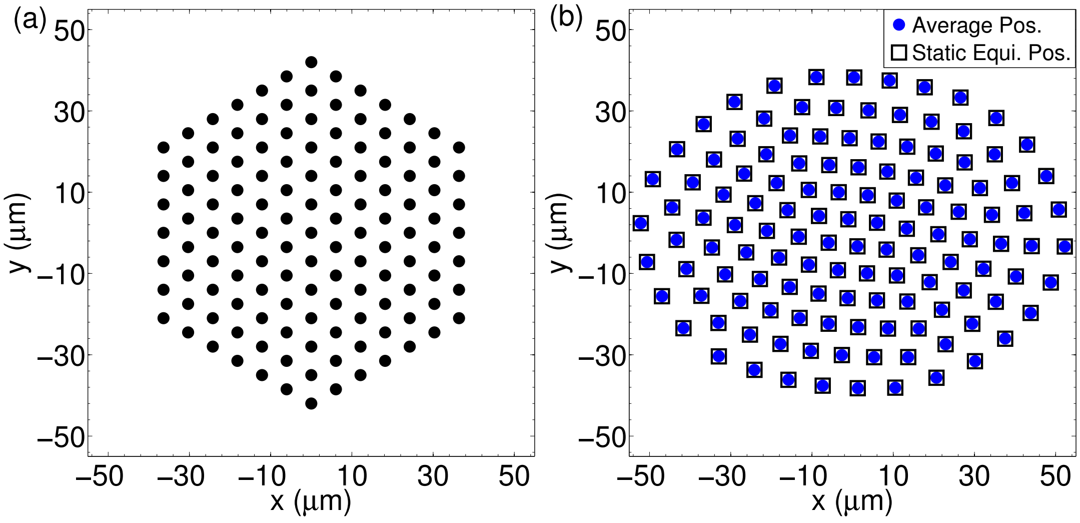

FIG. 1. (a) Initial configuration for ion crystal. 127 ions forming equilateral triangles with ion distance $7\,\mu m$ are arranged in a 2D hexagonal structure. (b) Stable ion configuration under the trap and Coulomb potential. Static equilibrium positions under the pseudopotential approximation are marked by (black) squares. Average ion positions found self-consistently by solving the Mathieu equations are marked by (blue) dots. The difference between two sets of equilibrium positions is around $0.03\,\mu m$ on average, which is hardly visible in the figure.

Note that the time-dependent part of the potential is isotropic in the coordinates, so it does not couple each Mathieu equations. We can find an orthogonal matrix Q that diagonalizes the first term, i.e. $Q(M_{DC}+M_C)Q^T=\Lambda$. Using the normal coordinates $\vec{s}=Q\vec{r}$, the equations of motion form decoupled Mathieu equations:

$$\frac{d^2 s_i}{d\xi^2}+(a_i-2q_i\cos(2\xi))s_i=f_i, \tag{7}$$

where $a_i=4\Lambda_{ii}/m\Omega_T^2$, $q_i=q=-4eV_0/md_0^2\Omega_T^2$, and $f_i=-\frac{4}{m\Omega_T^2}\,(Q\vec{g})_i$. The inhomogeneous Mathieu equations can be solved by substituting a special solution in the form of $s_i=f_i\sum_{n=0}^{\infty}c_i^{(n)}\cos(2n\xi)$, and the series coefficients $c_i^{(n)}$ can be computed numerically[2]. After that, the ion coordinates can be transformed back to the Cartesian coordinates $\vec{r}=Q^T\vec{s}$, where $\vec{r}$ can be expressed successively as

$$\vec{r}=\vec{r}^{\,(0)}+\vec{r}^{\,(1)}\cos(2\xi)+\vec{r}^{\,(2)}\cos(4\xi)+\cdots. \tag{8}$$

$\vec{r}^{\,(0)}$ now becomes the new average (equilibrium) positions, and can be substituted back to the expansion in equation (4). The ion positions $\vec{r}$ can be attained self-consistently in this manner. A dynamical expansion of the Coulomb potential around $\vec{r}^{\,(0)}+\vec{r}^{\,(1)}\cos(2\xi)$ may yield a more accurate result for the normal modes in the plane[3]. For our purpose, the static expansion is sufficient as we only need accurate ion positions $\vec{r}$ to compute the normal modes along the z direction. Numerically, we found that $\vec{r}^{\,(1)}\approx-\frac{q}{2}\vec{r}^{\,(0)}$ and $\vec{r}^{\,(2)}\approx\frac{q^2}{32}\vec{r}^{\,(0)}$, which are consistent with previous results[2,3]. Hence, micromotion only results in breathing oscillations about the average positions of each ion. The further the ion is from the center of the trap, the larger the amplitude of micromotion becomes.

Fig. 2 shows the amplitude of micromotion for each ion. The largest amplitude for the edge ion is around $1.35\,\mu$m, which is well below the ion separation ($7\sim10\,\mu$m), necessary for the formation of a well-defined crystal and for individual addressing.

II. NORMAL MODES ALONG THE TRANSVERSE DIRECTION

With the knowledge of the motion of ions in the x-y plane, we could find the normal modes and quantize the motion along the transverse (z) direction. As ions are confined in the plane, micromotion along the transverse direction is negligible. A harmonic pseudopotential is thus valid for the z direction. Expanding the Coulomb potential to second

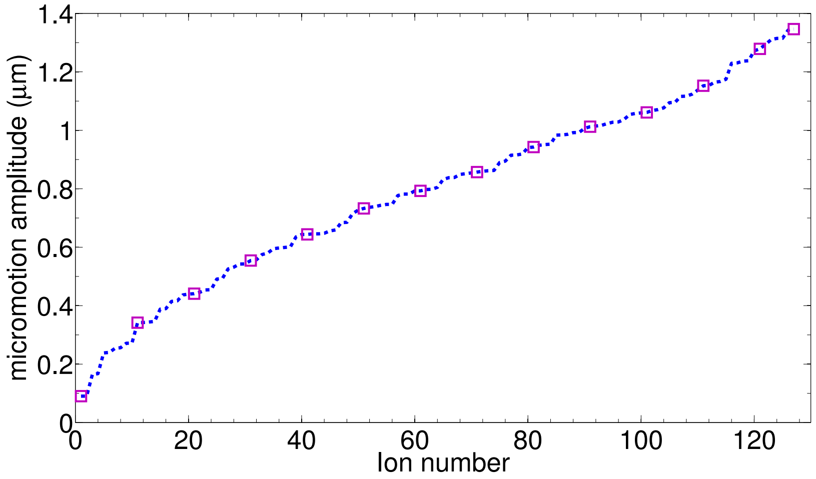

FIG. 2. Amplitude of micromotion for each ion (sorted in increasing order).

order again, we have

$$V_z = \frac{1}{2}m\omega_z^2 \sum_i z_i^2 + \frac{e^2}{4\pi\epsilon_0}\left[\sum_{i\neq j}\left(\frac{1}{r_{ij}^3}\right) z_i z_j - \sum_{i\neq j}\left(\frac{1}{r_{ij}^3}\right) z_i^2\right], \quad (9)$$

where $r_{ij} = \sqrt{(x_i - x_j)^2 + (y_i - y_j)^2}$. $x_i(t)$ and $y_i(t)$ are time-dependent though, due to the in-plane micromotion. From here, we can see explicitly that the transverse modes are decoupled from the planar modes. Expanding the term $1/r_{ij}^3(t)$ in series, one has

$$\frac{1}{r_{ij}^3} \approx \left\langle \frac{1}{r_{ij}^3} \right\rangle + M_{ij}\cos(\Omega_T t) + \cdots \quad (10)$$

The matrix element M_{ij} is in the order of $O(q)$ and can be obtained numerically from $\langle \cos(\Omega_T t)/r_{ij}^3 \rangle$. To have an intuitive understanding of the effect of micromotion on transverse modes, we take positions $\vec{r}$ in the form of Eq. (8), obtaining

$$\frac{1}{r_{ij}^3} \approx \left(\frac{1}{r_{ij}^{(0)}}\right)^3 \left(1 - \frac{q}{2}\cos(\Omega_T t) + \frac{q^2}{32}\cos(2\Omega_T t)\right)^{-3} + O(q^3), \quad (11)$$

where $r_{ij}^{(0)}$ is the zeroth order approximation using the average positions $\vec{r}^{\,(0)}$ without considering micromotion. Thus, $\langle 1/r_{ij}^3 \rangle \approx \left(1/r_{ij}^{(0)}\right)^3 (1 - 3q^2/4) + O(q^3)$, where we used the fact that $\langle \cos(\Omega_T t)\rangle = 0$ and $\langle \cos^2(\Omega_T t)\rangle = 1/2$. From the time-independent term $\langle 1/r_{ij}^3 \rangle$, we diagonalize V_z and find the normal modes as well as the eigenenergies in the transverse direction. Subsequently, we quantize the total Hamiltonian (with kinetic energy) and write $H = \sum_k \hbar\omega_k a_k^\dagger a_k$, where a_k is the annihilation operator for the quantized phonon mode, and ω_k is the corresponding eigenfrequency. In the interaction picture, $a_k \to a_k e^{-i\omega_k t}$. The time-dependent term containing $\cos(\Omega_T t)$ can then be treated as a perturbation; under the rotating wave approximation, since $\Omega_T \gg \omega_k$, the term affects the normal modes to the order of $O\left(q\omega_k^2/\Omega_T^2\right) \sim O(qq_z^2)$, which can be safely neglected. Since the first term in V_z is diagonal in z_i and the second term is reduced by a factor $(1 - 3q^2/4)$ by micromotion, the normal mode structure remains unchanged, and the mode frequencies are reduced slightly.

III. TWO-ION ENTANGLING GATE

The spin-dependent force on an ion is due to the AC Stark shift on each spin state. A different shift on the two internal spin states of an ion results in a Hamiltonian

$$H = \hbar\frac{|\Omega_{\text{eg}}|^2}{4\delta}\sigma^z, \quad (12)$$

where Ω_{eg} is the Rabi frequency of the laser beam and δ is the detuning from the excited state. By shining two laser beams at an angle with wave vectors $\mathbf{k}_1$, $\mathbf{k}_2$ and frequencies ω_1, ω_2, we have

$$\Omega_{\text{eg}} = \Omega_0 \left(e^{i(\mathbf{k}_1 \cdot \mathbf{r} + \omega_1 t + \phi)} + e^{i(\mathbf{k}_2 \cdot \mathbf{r} + \omega_2 t)} \right), \tag{13}$$

where ϕ is the phase difference between two beams. So we have

$$H = \hbar\Omega \left(1 + \cos(\Delta k \cdot z + \mu t + \phi)\right) \sigma^z, \tag{14}$$

where $\Omega = \Omega_0^2/2\delta$ is the effective two-photon Rabi frequency, $\Delta k \hat{z} = \mathbf{k}_1 - \mathbf{k}_2$ is aligned along the z direction, and $\mu = \omega_1 - \omega_2$. As we are mostly interested in the two-qubit entangling gate, which is the building block for universal quantum gates, we consider laser beams shining on two ions, and ignore the first term $\hbar\Omega\sigma^z$ in the Hamiltonian that only induces single bit operations. We therefore have

$$H = \sum_{j=1}^{2} \hbar\Omega_j \cos(\Delta k \cdot z_j + \mu t + \phi_j)\sigma_j^z, \tag{15}$$

The ion position $z_j = z_{j0} + \delta z_j$, where z_{j0} is the equilibrium position and δz_j is the small displacement. We dump the term $\Delta k \cdot z_{j0}$ to the phase ϕ_j, and expand the cosine term in the Lamb-Dicke limit $\Delta k \cdot \delta z_j \ll 1$,

$$\begin{aligned}
H &= \sum_{j=1}^{2} \hbar\Omega_j \cos(\Delta k \cdot \delta z_j + \mu t + \phi_j)\sigma_j^z & (16)\\
&\approx -\sum_{j=1}^{2} \hbar\Omega_j \sin(\Delta k \cdot \delta z_j) \sin(\mu t + \phi_j)\sigma_j^z & (17)\\
&\approx -\sum_{j,k} \hbar\Omega_j \sin(\mu t + \phi_j)\Delta k \left[\sqrt{\frac{\hbar}{2m\omega_k}} b_j^k a_k^\dagger + \text{H.c.} \right] \sigma_j^z & \\
&= -\sum_{j=1}^{2} \sum_k \chi_j(t) g_j^k (a_k^\dagger + a_k)\sigma_j^z & (18)
\end{aligned}$$

In step (17), we drop the cosine-cosine term $\hbar\Omega_j \cos(\Delta k \cdot \delta z_j) \cos(\mu t + \phi_j)\sigma_j^z \approx \hbar\Omega_j \cos(\mu t + \phi_j)\sigma_j^z$ since $\Delta k \cdot \delta z_j \ll 1$ and it thus does not couple the phonon modes to the spin (in the first-order approximation), resulting in a single-qubit operation. Various terms are defined as

$$\delta z_j = \sum_k \sqrt{\frac{\hbar}{2m\omega_k}} b_j^k a_k^\dagger + \text{H.c.} \tag{19}$$

where b_j^k are the mode vector for mode k, $a_k^\dagger$ creates the k-th phonon mode (harmonic oscillator mode). The matrix b_n^k diagonalizes the approximate harmonic potential of the system.

$$\chi_j(t) = \hbar\Omega_j \sin(\mu t + \phi_j) \tag{20}$$

$$g_j^k = \eta_k b_j^k, \quad \text{where} \quad \eta_k = \Delta k \sqrt{\frac{\hbar}{2m\omega_k}} \tag{21}$$

η_k is the Lamb-Dicke parameter, $\eta_k \ll 1$ to be valid (for the expansion). For $\Delta k = 8\mu m^{-1}$, $m = 171u$ for Ytterbium, and take the transverse mode $\omega_k = 2\pi \times 2\text{MHz}$. We will have $\eta_k \approx 0.03$. Going into the interaction picture and replacing $a_k \to a_k e^{-i\omega_k t}$, we have

$$H_{\text{I}} = -\sum_{j=1}^{2} \sum_k \chi_j(t) g_j^k (a_k^\dagger e^{i\omega_k t} + a_k e^{-i\omega_k t})\sigma_j^z \tag{22}$$

The evolution operator can be obtained from the Hamiltonian as[4,5]

$$U(\tau) = \exp\big(i\sum_j \phi_j(\tau)\sigma_j^z + i\sum_{j<n}\phi_{jn}(\tau)\sigma_j^z\sigma_n^z\big), \tag{23}$$

$$\phi_j(\tau) = -i\sum_k \alpha_j^k(\tau)a_k^\dagger - \alpha_j^{k*}(\tau)a_k \tag{24}$$

$$\alpha_j^k(\tau) = \frac{i}{\hbar}g_j^k\int_0^\tau \chi_j(t)e^{i\omega_k t}dt, \tag{25}$$

$$\phi_{jn}(\tau) = \frac{2}{\hbar^2}\sum_k g_j^k g_n^k \int_0^\tau\int_0^{t_2}\chi_j(t_2)\chi_n(t_1)\times\sin(\omega_k(t_2-t_1))dt_1dt_2. \tag{26}$$

To obtain a two-qubit entangling gate, we need $\alpha_j^k = 0$ so that the spin and phonons are disentangled at the end of the gate, and $\phi_{jn}(\tau) = \pi/4$. This is the starting point to calculate the fidelity of the gate.

[1] McLachlan, N. W. *Theory and application of Mathieu functions* (Clarendon Press, 1951).
[2] Shen, C. & Duan, L.-M. High-fidelity quantum gates for trapped ions under micromotion. *Phys. Rev. A* **90**, 022332 (2014).
[3] Landa, H., Drewsen, M., Reznik, B. & Retzker, A. Modes of oscillation in radiofrequency paul traps. *New J. Phys.* **14**, 093023 (2012).
[4] Zhu, S.-L., Monroe, C. & Duan, L.-M. Trapped ion quantum computation with transverse phonon modes. *Phys. Rev. Lett.* **97**, 050505 (2006).
[5] Kim, K. *et al.* Entanglement and tunable spin-spin couplings between trapped ions using multiple transverse modes. *Phys. Rev. Lett.* **103**, 120502 (2009).
